# Supplementary material for: Cerebellar implementation of movement sequences through feedback
Source: eLife. 2018 Jul 31;7:e37443. doi: 10.7554/eLife.37443 (PMC6107335; doi:10.7554/eLife.37443)
Supplement: Figure 7—source data 1. [file elife-37443-fig7-data1.docx]

A)

|  | Latency to criterion versus latency to criterion | Peak time versus onset time |
| --- | --- | --- |
| Second CR versus first CR | N = 1163 trials, P = 0.016 | N = 1142 trials, P = 0.0033 |
| Third CR versus second CR | N = 126 trials, P = 0.01 | N = 126 trials, P < 0.001 |
| Fourth CR versus third CR | N = 14 trials, P = 0.28 | N = 14 trials, P = 0.14 |

B)

|  | Left CR latency to criterion versus right CR latency to criterion | Left CR latency to criterion versus right CR half-peak time |
| --- | --- | --- |
| Δ = 300 ms | N = 226 trials, P < 0.001 | N = 223 trials, P = 0.0013 |
| Δ = 400 ms | N = 605 trials, P = 0.0021 | N = 599 trials, P = 0.0016 |
| Δ = 500 ms | N = 777 trials, P = 0.043 | N = 774 trials, P = 0.015 |

Summary of Kolmogorov-Smirnov test results related to Figure 7 (real versus shuffled distributions of inter-CR timing).

**(A)** Results for data from subjects trained in ipsilateral sequence. Table rows and color indicate for which pair of CRs the results are shown, columns indicate the pair of CR timing measures between which inter-CR timing distribution was calculated. The table shown results of Kolmogorov-Smirnov test comparing real distributions of inter-CR timing and distribution with first CR timing permuted across trials (Figure 7, **C, E**). **(B)** Similar format of results for data from contralateral sequence (Figure 7, **D, F**). Here rows and colors indicate the used gap interval.
